# Prognostic significance of kynurenine 3-monooxygenase and effects on proliferation, migration, and invasion of human hepatocellular carcinoma

**DOI:** 10.1038/srep10466

**Published:** 2015-06-23

**Authors:** Haojie Jin, Yurong Zhang, Haiyan You, Xuemei Tao, Cun Wang, Guangzhi Jin, Ning Wang, Haoyu Ruan, Dishui Gu, Xisong Huo, Wenming Cong, Wenxin Qin

**Affiliations:** 1 State Key Laboratory of Oncogenes and Related Genes, Shanghai Cancer Institute, Renji Hospital, Shanghai Jiao Tong University School of Medicine; 2 Department of Pathology, Eastern Hepatobiliary Surgery Hospital, Second Military Medical University

## Abstract

Kynurenine 3-monooxygenase (KMO) is a pivotal enzyme in the kynurenine pathway of tryptophan degradation and plays a critical role in Huntington’s and Alzheimer’s diseases. This study aimed to examine the expression of KMO in human hepatocellular carcinoma (HCC) and investigate the relationship between its expression and prognosis of HCC patients. We first analyzed KMO expression in 120 paired HCC samples (HCC tissues *vs* matched adjacent non-cancerous liver tissues), and 205 clinical HCC specimens using immunohistochemistry (IHC). Kaplan-Meier survival and Cox regression analyses were executed to evaluate the prognosis of HCC. The results of IHC analysis showed that KMO expression was significantly higher in HCC tissues than that in normal liver tissues (all p < 0.05). Survival and recurrence analyses showed that KMO was an independent prognostic factor for overall survival (OS) and time to recurrence (TTR) (both p<0.01). And *in vitro* studies revealed that KMO positively regulated proliferation, migration, and invasion of HCC cells. These results suggest that KMO exhibits tumor-promoting effects towards HCC and it may serve as a novel prognostic marker in HCC.

Hepatocellular carcinoma (HCC) is the fifth most prevalent cancer and the third major cause of cancer-related death in the world^1^. Despite many advances in HCC therapy such as surgery, chemotherapy and biologics, majority of HCC patients still has a poor prognosis due to high frequency of metastasis and recurrence^2^,^3^. It has been reported that the 5-year survival rate of HCC patients is as low as 25–39%, and its recurrence rate remains about 80%^4^,^5^. Therefore, it is critical to understand the etiology, illustrate the mechanisms underlying HCC initiation and progression, and further identify valuable factors for prognosis prediction and novel therapeutic strategies.

Kynurenine 3-monooxygenase (KMO), a pivotal enzyme in the kynurenine pathway (KP) of tryptophan degradation, has been suggested to play a critical role in Huntington’s (HD) and Alzheimer’s diseases (AD)^6^,^7^,^8^. It is widely distributed in peripheral tissues, including liver and kidney, and in phagocytes such as macrophages and monocytes^9^,^10^, and also in microglial cells in central nervous system^11^,^12^. As a FAD-dependent enzyme, KMO localizes to the outer mitochondrial membrane and controls the synthesis of several KP metabolites, including 3-hydroxykynurenine (3-HK), quinolinic acid (QUIN), and kynurenicacid (KYNA), as well as anthranilic acid. These bioactive metabolites were found to frequently associate with brain disorders^8^, peripheral inflammatory conditions^13^, and cancer^14^,^15^. However, whether KMO deregulation also occurs in human HCC remains unclear. In this study, we investigated the expression of KMO, evaluated its prognostic significance, and explored the role of KMO in HCC. Our data indicate that KMO is remarkably increased in HCC and can be served as a promising biomarker of HCC prognosis.

## Materials and Methods

### Patients and Specimens

Paraffin-embedded pathological specimens in prognostic groups were obtained from the archives of the Eastern Hepatobiliary Hospital (EHBH) between 1996 and 2001, and followed until October 2010. No patients in this study received sorafenib treatment. Tumor stage was defined according to the American Joint Committee on Cancer (AJCC 2010, 7th edition) TNM staging system^16^. The grade of tumor differentiation was assigned by the Edmondson-Steiner grading system. Micrometastases were defined as tumors adjacent to the border of the main tumor that was only observed under the microscope^17^. Then, 205 and 182 HCC patients were randomly selected from this cohort as the study population and reviewed retrospectively.

Patient follow-up was performed every 2–3 months during the first year after surgery and 3–6 months thereafter until October 2010. The median follow-up was 40.8 month (range, 0.3–141 month). All follow-up examinations were performed by two physicians unaware of the study. All patients were monitored by abdomen ultrasonography, chest X-ray, and a test for the serum AFP concentration every month during the first year after surgery and every 3–6 months thereafter. A computed tomography scan or magnetic resonance imaging of the abdomen was performed every 6 months or immediately after a recurrence was suspected. The diagnosis criteria for recurrence were equal to that for the preoperative diagnosis. The overall survival (OS) was defined as the length of time between the surgery and death or the last follow-up examination. The time to recurrence (TTR) was calculated from the date of tumor resection until the detection of tumor recurrence, death or the last observation.

An additional 50 HCC patients as test cohort were randomly recruited between March 13, 2000 and January 31, 2002 for immunohistochemistry (IHC) analysis. These resected samples were also subjected to western blot verification (n = 10). To verify immunohistochemical results of test cohort, another large-scale cohort as validation cohort, including 70 cases randomly recruited between February 18, 2002 and March 6, 2003, was analyzed via IHC. In addition, 43 HCC tissue samples and 43 liver cirrhosis tissue samples were randomly collected between February 18, 2002 and March 6, 2003, and used for further IHC expression analysis.

For the use of clinical materials for research purposes, prior patients' consents and approval were obtained from the Ethics Committee of Renji Hospital, Shanghai Jiao Tong University School of Medicine and EHBH of the Second Military Medical University. All experiments were performed in accordance with approved guidelines of Shanghai Jiao Tong University School of Medicine.

### Immunohistochemistry and Scoring

Immunohistochemistry, signal evaluation, and integrated optical density (IOD) analysis were performed as described previously^18^. TDO antibody was purchased from Aviva Systems Biology (1:100) and KMO antibody was purchased from LifeSpan BioSciences (1:100). Photographs of three representative fields were captured under high-power magnification (×200); identical settings were used for each photograph. IOD was counted and measured using Image-Pro Plus v6.0 software, and mean IOD was calculated from three photographs per specimen.

Immunostaining scores were independently evaluated by two pathologists who were blinded to the clinical outcome. Semiquantitative scores were used to analyze immunostaining of each HCC case in tissue microarray. Intensity of staining was categorized into −, +, ++, or +++, denoting negative, weak, moderate, or strong staining, respectively. Extent of immunostaining was categorized into 0 (0–5%), 1 (6–25%), 2 (26–50%), or 3 (>51%) on the basis of the percentage of positive cells. Three random microscope fields per tissue were calculated. The sum of intensity and extent of staining was used as final score of expression level, and determined by the formula: final score = intensity score × percentage score. The final score of ≤3 was defined as low expression, and >3 as high expression.

### Cell Culture and Transfection

Human normal liver cell lines L02 and SMMC7721 were obtained from Shanghai Institute of Cell Biology, Chinese Academy of Sciences. SK-Hep1 and PLC-PRF5 were purchased from the American Type Culture Collection. MHCC97H, MHCC97L, and HCCLM3 were provided by the Liver Cancer Institute of Zhongshan Hospital of Fudan University (Shanghai, China). Huh7 cells were purchased from Riken Cell Bank (Tsukuba, Japan). All the cell lines were cultured in Dulbecco’s modified Eagle’s medium (DMEM) (Gibco, Gaithersburg, MD, USA) containing 10% fetal bovine serum (FBS), 100 mg/ml penicillin, and 100 mg/ml streptomycin. All of the cells were incubated in a humidified atmosphere of 5% CO_2_ and 95% air at 37 °C. SK-Hep1 and MHCC-97H cells were transfected with KMO siRNA using lipofectamine 2000 (Invitrogen, Carlsbad, CA) according to the manufacturer’s instructions. SMMC7721 and Huh7 were transfected with the vector constitutively expressing KMO(pCMV6-XL5/KMO), or the control empty vector (pCMV6-XL5/Vector) according to the manufacturer’s instructions. The siRNA duplexes targeted KMO (siRNA#1: forward 5’-CCAAGGUAUUCCCAUGAGATT-3’, reverse 5’-UCUCAUGGGAAUACCUU- GGTT-3’; siRNA#2: forward 5’-CAGCCCAUGAUAUCUGUAATT-3’, reverse 5’-UUACAGAUAUCAUGGGCUGTT-3’) and scramble siRNA duplex (forward: 5’-UUCUCCGAACGUGUCACGUTT-3’; reverse: 5’-ACGUGACACGUUCGGA- GAATT-3’) were chemically synthesized by Biomics Biotechnologies Co. Ltd (Shanghai, China). The pCMV6-XL5/KMO and pCMV6-XL5/Vector were purchased from Origene (Rockville, MD).

### RNA isolation and Real Time PCR

Total RNA was extracted using TRIzol reagent (Invitrogen) and reversely transcribed using PrimeScript™ RT Reagent Kit (TaKaRa Biotechnology). Real time polymerase chain reaction (Real Time PCR) was subsequently performed following the manual (TaKaRa Biotechnology). Expression levels were normalized against β-actin, and relative expression levels were displayed using 2-^ΔΔCt^ method. Primer sequences used are as follows: KMO: Forward:TGCTGAGAAATACCCCAATGTG; Reverse: CTGACAGTTGAATAG

GCTCCATC; β-actin: Forward: TTGTTACAGGAAGTCCCTTGCC; Reverse: ATGCTATCACCTCCCCTGTGTG.

### Western Blot Analysis

Briefly, tissue and cell samples were homogenated in a RIPA buffer (Qiagen, China). After centrifugation at 12,000 g, 4 °C for 30 min, 50 μg of protein samples were fractionated by sodium dodecyl sulfate polyacrylamide gel electrophoresis (SDS-PAGE) and transferred to nitrocellulose membranes. After blocking non-specific binding sites for 60 min with 5 % non-fat milk, the membranes were incubated with rabbit monoclonal antibody against KMO (1:1,800; LifeSpan BioSciences, Inc.), or β-actin (1:1,000;Santa Cruz) at 4 °C overnight, respectively, and subsequently, probed with HRP-conjugated anti-rabbit secondary antibody (1:5,000; Santa Cruz) for 45 min at room temperature. Chemiluminescence detection was performed using SuperSignal West Femto Maximum Sensitivity Substrate Kit 19 (Pierce). Membranes were exposed and recorded with Molecular Imager ChemiDoc XRS+System (Bio-Rad, CA, USA).

### Cell Proliferation Assay

Cell proliferation was measured using the Cell Counting Kit-8 reagent (CCK-8, Dojindo, Japan). Cells were seeded into a 96-well plate at 2 ×10^3^ cells per well with 100 μl complete medium and cultured at 37 °C. 10 μl CCK-8 solution was added to each well after 0, 24, 48 and 72 hours, respectively.

### Migration Assay

Cell migration assays were performed using Transwell filter champers (BD Biosciences). 5 × 10^4^ cells in 200 μl serum-free DMEM were seeded in the upper chamber of a transwell and 800 μl medium supplemented with 15% FBS was added to the lower chamber. After indicated time (12 hours for SK-Hep1, 24 hours for MHCC-97H, 20 hours for SMMC7721 and 20 hours for Huh7) of incubation at 37 °C, migrated cells were fixed and stained with a dye solution containing 0.1% crystal violet and 20% methanol. Cells adhering to the lower side of the inserts were counted and imaged through an IX71 inverted microscope (Olympus). Five random microscopic fields were counted per well for each group, and the experiments were repeated at least three times independently.

### Invasion Assay

For *in vitro* invasion assay, transwell filter champers (BD Biosciences) and transwells coated with Matrigel (BD Biosciences) were utilized according to manufacturer’s instructions. 5 × 10^4^ cells in 200 μl serum-free DMEM were seeded in the upper chamber of a transwell and 800 μl medium supplemented with 15% FBS was added to the lower chamber. After indicated time (20 hours for SK-Hep1, 40 hours for MHCC-97H, 24 hours for SMMC7721 and 30 hours for Huh7) of incubation at 37 °C, invaded cells were fixed and stained with a dye solution containing 0.1% crystal violet and 20% methanol. Cells adhering to the lower side of the inserts were counted and imaged through an IX71 inverted microscope (Olympus). Five random microscopic fields were counted per well for each group, and the experiments were repeated at least three times independently.

### Statistic Analysis

Differences among variables were assessed by χ^2^ analysis or two-tailed Student t test. Kaplan-Meier analysis was used to assess survival. Log-rank tests were used to compare survival of patients between subgroups. Multivariate analyses were performed by multivariate Cox proportional hazard regression model. The experiments were performed in triplicates and data were presented as mean ± SEM. Differences were considered to be statistically significant for p < 0.05.

## Results

### Up-regulation of KMO in HCC tissues

**Figure 1:**
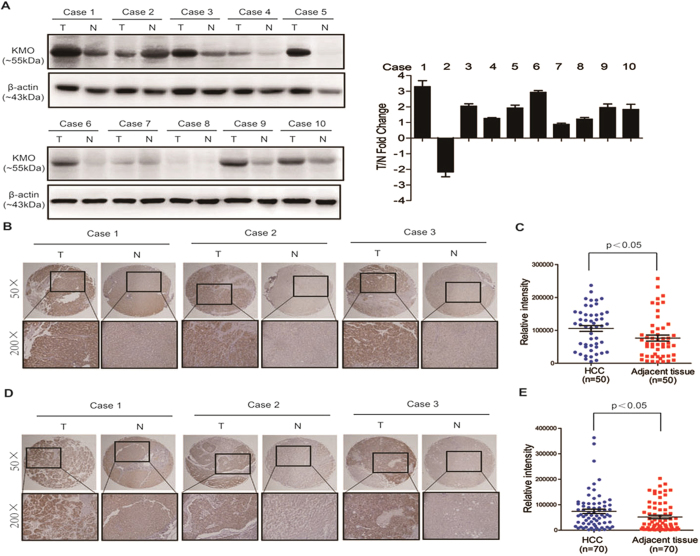
Up-regulation of KMO in HCC tissues. **(A)** Western blotting analysis for KMO in 10 paired samples of HCC tissues (T) and matched adjacent non-tumorous liver tissues (N). *Left panel* Representative Western blots for KMO and β-actin were shown. *Right panel* Band intensities of KMO were normalized to β-actin and showed as T/N Fold Change. Uncropped full-length blots for A are shown in the Supplementary Fig. 1. **(B)** IHC staining of KMO in 50 pairs of HCC tissues (T) and matched adjacent non-tumorous liver tissues (N) (Test cohort). **(C)** Integrated optical density (IOD) for KMO was obtained from the test cohort. **(D)** IHC staining of KMO in 70 pairs of HCC tissues (T) and matched adjacent non--tumorous liver tissues (N) (Validation cohort). **(E)** IOD for KMO was obtained from the validation cohort.

KMO expression was first analyzed in 10 matched pairs of HCC and adjacent non-tumorous liver tissue by Western blotting. As shown in Fig. 1A, in most cases, KMO expression in HCC tissue was obviously higher than in adjacent non-tumorous liver tissue of the same HCC patient. We next performed IHC analysis for KMO using a tissue microarray as a test cohort, which contained 50 paired HCC tissue samples. Immunohistochemical results showed the staining density of KMO in HCC group was obviously stronger than that in adjacent non-tumorous liver tissue group (Fig. 1B,C, p < 0.05). We further analyzed KMO expression in another independent validation cohort of 70 HCC patients by IHC. Similarly, KMO expression was significantly increased in HCC group compared with adjacent non-tumorous liver tissue group (Fig. 1D,E, p < 0.05).

**Figure 2:**
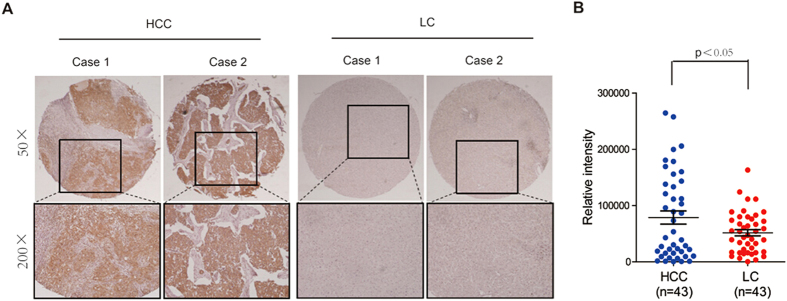
Comparison of KMO levels in HCC patients and LC patients. **(A)** IHC staining of KMO in 43 HCC patients and 43 LC patients. **(B)** IOD for KMO was obtained from (**A**).

We also performed IHC analysis for KMO using a tissue microarray, which contained 43 tumor tissues from HCC patients and 43 liver tissues from liver cirrhosis patients. Our data showed that KMO expression exhibited a progressive increase from liver cirrhosis to HCC (Fig. 2A,B, p < 0.05).

### Association of KMO expression with clinicopathologic features in HCC patients

Next, we investigated relationship between KMO expression and clinicopathological variables of HCC patients. IHC was performed to assess KMO expression in a retrospective cohort with 205 HCC patients, including 59 cases of stage I (28.8%), 120 cases of stage II (58.5%), and 26 cases of stage III (12.7%), based on the TNM staging. KMO expression in the 205 samples was determined as high expression in 70 cases (70/205, 34.1%) and low expression in 135 cases (135/205, 65.9%). Spearman analysis revealed significant correlation between KMO and tumor differentiation (p = 0.004). However, there was no significant association between expression of KMO and other clinicopathological parameters such as age, sex, HBsAg status, tumor size, Child-Pugh, and vascular invasion (Table 1).

### Correlation of KMO expression with prognosis of HCC patients

**Figure 3:**
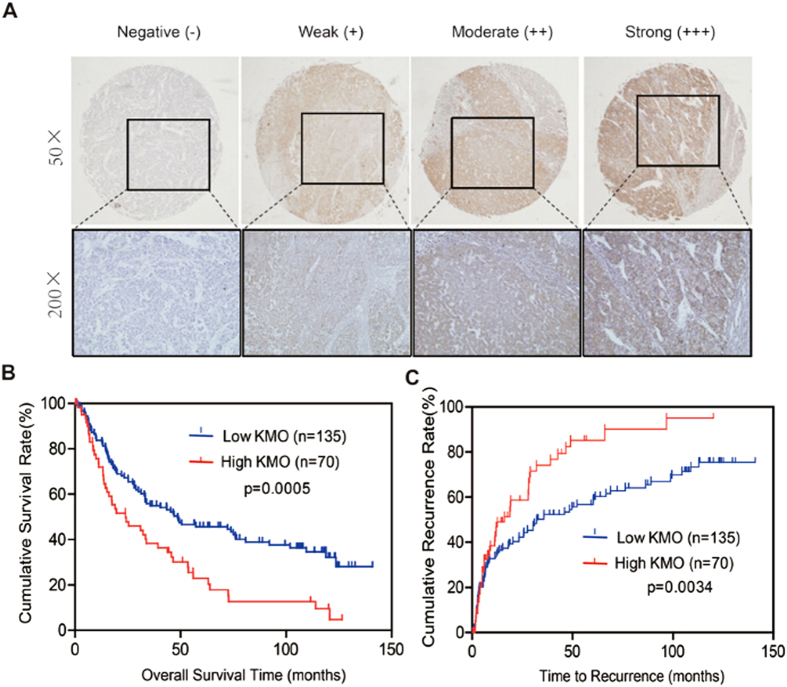
Correlation of KMO expression with OS and TTR in HCC patients. **(A)** Representative photomicrographs showed negative (−), weak (+), moderate (++), or strong (+++) immunostaining of KMO in HCC specimens (magnification, × 50, × 200). **(B, C)** Kaplan-Meier curves of OS **(B)** and TTR **(C)** in 205 HCC patients.

To determine the value of KMO for the prognosis of postsurgical HCC patients, Kaplan-Meier overall survival (OS) and time to recurrence (TTR) analyses were conducted. At the time of last follow-up, among the 205 HCC patients in the cohort, 118 had tumor recurrence and 120 had died. The mean OS was 48.7 months for patients with low KMO expression and 24.0 months for patients with high KMO expression. The mean TTR was 32.3 months for patients with low KMO expression and 16.2 months for patients with high KMO expression. These results indicated that patients with high KMO expression had much shorter OS times (Fig. 3A, p = 0.0005) and a higher tendency of disease recurrence (Fig. 3B, p = 0.0034). Moreover, we also performed IHC analysis for tryptophan 2,3-dioxygenase (TDO), which is the main KP enzyme in liver cells, using a tissue microarray with 182 HCC patients. Kaplan-Meier OS and TTR analyses were conducted and results showed that TDO had no prognostic value for postsurgical HCC patients (Supplementary Fig. 2).

**Figure 4:**
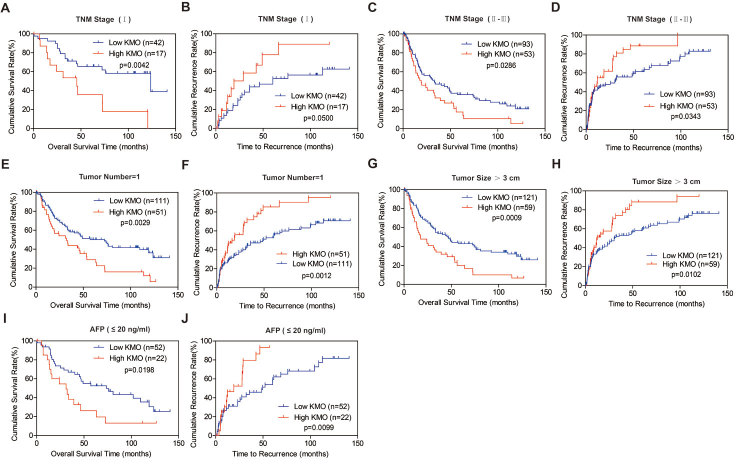
Correlation of KMO expression with OS and TTR in HCC subgroups. Analyses of OS and TTR by TNM stage **(A–D)**, tumor number = 1 **(E, F)**, Tumor Size >3 cm **(G, H)**, and AFP ≤ 20 ng/ml **(I, J)**.

Additionally, prognostic value of KMO was further confirmed by stratified OS and TTR analyses. Results showed that high expression of KMO was closely connected with OS and TTR after surgical resection in subgroups including TNM stage (TNM stage I , Fig. 4A,B; TNM stage II-III, Fig. 4C,D), tumor number (tumor number = 1, Fig. 4E,F), tumor size (tumor size >3 cm, Fig. 4G,H), and AFP concentration (AFP ≤20 ng/ml, Fig. 4I,J).

### Univariate and multivariate analyses of prognostic variables in HCC patients

We next evaluated prognostic significance of KMO and other clinicopathologic parameters in HCC using univariate analysis. As shown in Table 2, KMO as well as TNM stage, tumor number, tumor differentiation, and microvascular invasion, was responsible for the OS and TTR of HCC patients.

Multiple Cox regression analysis was further utilized to evaluate independent prognostic value of KMO. Results revealed that KMO was an independent prognostic marker for OS (HR: 1.700, 95% CI: 1.161–2.489, p = 0.006) and TTR (HR: 1.763, 95% CI: 1.193–2.606, p = 0.004) in HCC patients (Table 2).

### Inhibition of cell proliferation, migration, and invasion by KMO knockdown

**Figure 5:**
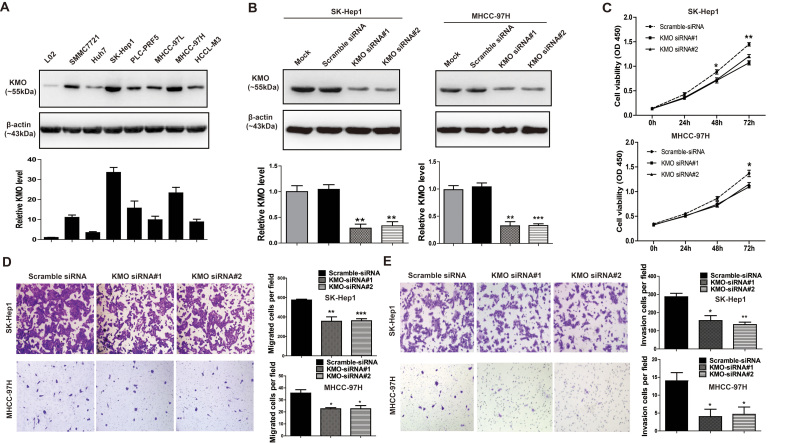
Inhibition of proliferation, migration, and invasion by KMO siRNAs *in vitro*. **(A)** Analysis of KMO expression in HCC cell lines by Western blotting. *Upper panel* Representative Western blots for KMO and β-actin were shown. *Lower panel* Band intensities of KMO were normalized to β-actin and showed as Relative Intensity to L02 cells. Uncropped full-length blots were showed in the Supplementary Fig. 3. **(B)** SK-Hep1 and MHCC-97H cells were transfected with no siRNA (Mock), siRNA control (Scramble siRNA) or siRNAs against KMO (KMO siRNA#1 and KMO siRNA#2), respectively. Knockdown efficiency of KMO was verified by Western blotting. *Upper panel* Representative Western blots for KMO and β-actin were shown. *Lower panel* Band intensities of KMO were normalized to β-actin and showed as Relative Intensity to Mock cells, respectively. Uncropped full-length blots were showed in the Supplementary Fig. 5. **(C)** Cell proliferation of SK-Hep1 and MHCC-97H cells transfected with KMO siRNA was observed by CCK8 assay. **(D)** Cell migration of SK-Hep1 and MHCC-97H cells transfected with KMO siRNA was measured by transwell migration assays. **(E)** Cell invasion of SK-Hep1 and MHCC-97H cells transfected with KMO siRNA was measured by matrigel invasion assays. Data are mean ± SD of 3 biological replicates (*p < 0.05, **p < 0.01, ***p < 0.001).

We also assessed protein level of KMO in a normal liver cell line L02 and seven HCC cell lines, including SMMC7721, Huh7, SK-Hep1, PLC-PRF5, MHCC-97L, MHCC-97H, and HCC-LM3. Results showed that KMO expression was much higher in HCC cell lines, compared with the normal liver cell line L02 (Fig. 5A). Then, KMO was knocked down in SK-Hep1 and MHCC-97H cells, which expressed high levels of KMO, by small interfering RNA (siRNA). The mRNA and protein levels of KMO were effectively down-regulated by two KMO siRNAs at 48 hours of posttransfection (Supplementary Fig. 4 and Fig. 5B). As indicated by results of CCK8 assays (Fig. 5C), the proliferation of SK-Hep1 cells was significantly inhibited after KMO siRNAs transfection on day 2 (p < 0.05) and day 3 (p < 0.01). Likewise, compared to scramble siRNA transfected cells, proliferation of MHCC-97H cells also significantly inhibited after KMO siRNAs transfection on day 3 (p < 0.05). Besides, effects of KMO on cell migration and invasion were also investigated using transwell migration assays and matrigel invasion assays, respectively. Our results showed that KMO knockdown significantly decreased the migration and invasion rates of both SK-Hep1 and MHCC-97H cells *in vitro*, respectively (Fig. 5D,E, all p < 0.05).

### Enhancement of cell proliferation, migration, and invasion by KMO over-expression

**Figure 6:**
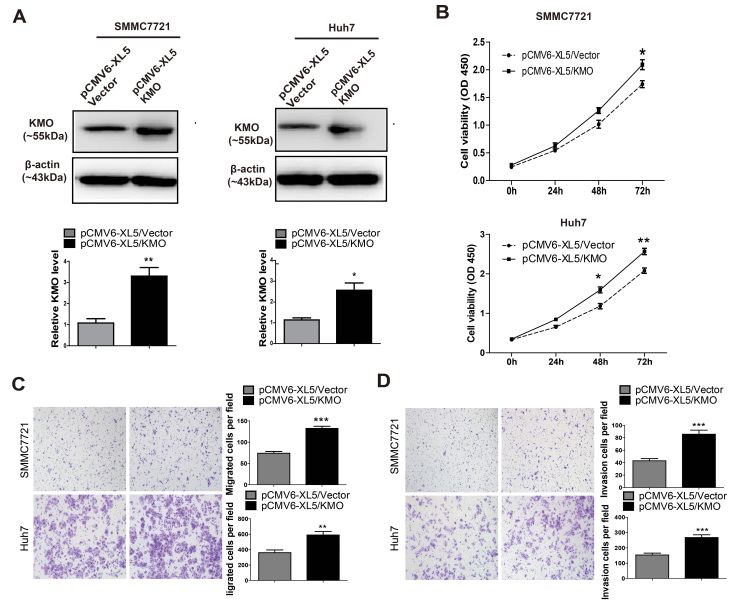
Enhancement of proliferation, migration, and invasion by KMO over-expression *in vitro*. (**A**) SMMC7721 and Huh7 cells were transfected with a vector constitutively expressing KMO (pCMV6-XL5/KMO) and a empty control vector (pCMV6-XL5/Vector), respectively. KMO expression was detected by Western blotting. *Upper panel* Representative Western blots for KMO and β-actin were shown. *Lower panel* Band intensities of KMO were normalized to β-actin and showed as Relative Intensity to pCMV6-XL5/Vector cells. Uncropped full-length blots were showed in the Supplementary Fig. 7 (**B**) Cell proliferation of SMMC7721 and Huh7 cells transfected with pCMV6-XL5/Vector and pCMV6-XL5/KMO were observed by CCK8 assay. (**C**) Cell migration of SMMC7721 and Huh7 cells transfected with pCMV6-XL5/Vector and pCMV6-XL5/KMO was measured by transwell migration assays. (**D**) Cell invasion of SMMC7721 and Huh7 cells transfected with pCMV6-XL5/Vector and pCMV6-XL5/KMO was measured by matrigel invasion assays. Data are mean ±SD of 3 biological replicates (*p < 0.05, **p < 0.01, ***p < 0.001).

To further confirm the effects of KMO on proliferation, migration, and invasion, SMMC7721 and Huh7 cells were transfected with a vector constitutively expressing KMO (pCMV6-XL5/KMO) and a empty control vector (pCMV6-XL5/Vector), respectively. The mRNA and protein levels of KMO were significantly increased in pCMV6-XL5/KMO cells compared with pCMV6-XL5/Vector (Supplementary Fig. 6 and Fig. 6A). As indicated by results of CCK8 assays (Fig. 6B), the proliferation of SMMC7721 cells was significantly increased after KMO over-expression on day 3 (p < 0.05). Likewise, compared to empty control vector transfected cells, proliferation of Huh7 cells significantly increased after KMO over-expression on day 2 (p < 0.05) and day 3 (p < 0.01). Furthermore, KMO over-expression also significantly increased the migration and invasion rates of both SMMC7721 and Huh7 cells *in vitro*, respectively (Fig. 6C,D, all p < 0.01).

## Discussion

Despite substantial advances in surgery and chemotherapy for HCC in the past time^3^,^19^,^20^. therapeutic failure and mortality are still very common. The current pathological TNM (pTNM) stage and histological grading systems are established and can indicate HCC prognosis to a certain extent. However, due to tumor heterogeneity and accumulation of genetic and epigenetic alterations, patients with the same pTNM stage and/or histological grade of HCC often demonstrate considerable variability in tumor recurrence and metastasis^21^. Thus, searching for valuable diagnostic and prognostic predictors that can effectively distinguish between patients with favorable or unfavorable prognoses in the same stage and/or grade are urgently needed. Although previous studies have suggested several molecular biomarkers of HCC^22^,^23^,^24^,^25^,^26^, the novel biomarkers that can identify tumor recurrence and aid risk assessment remain substantially limited.

In the present study, we provide the first evidence that increased expression of KMO is correlated with an unfavorable clinical outcome of HCC patients. IHC staining in two independent cohorts (test cohort: 50 paired; validation cohort: 70 paired) indicated that KMO was abnormally elevated in HCC specimen compared with adjacent non-tumorous liver tissue. In a cohort of 205 HCC patients, Kaplan-Meier OS and TTR analyses showed that high KMO expression was associated with short HCC recurrence and poor prognosis after surgical resection. Multiple Cox regression analysis further conformed that KMO was an independent prognostic marker for OS and TTR. In addition, our data also showed a progressive increase of KMO from liver cirrhosis to HCC. These findings implicate that up-regulation of KMO may be a common feature in HCC and it can serve as an independent prognostic marker to identify patients with poor clinical outcome.

Identification of novel and specific prognostic biomarker for patients with early stage HCC is remarkably important^27^. Although AFP is most commonly employed and currently available as serological markers of HCC for surveillance, diagnosis and patient outcome prediction^28^,^29^,^30^, HCC patients without AFP elevation (AFP < 20 ng/ml) are missed and subsequently progress to late stage HCC before becoming clinically symptomatic and detectable^28^,^30^. Moreover, the diagnostic and prognostic sensitivity of AFP was poor in the early stage of HCC, especially when used alone. Here, we evaluated the clinical usefulness of KMO as a prognostic factor in HCC patients with AFP < 20 ng/ml, which accounted for 36.1% of HCC patients in our study cohort. In addition, our results also showed that high expression of KMO in HCC patients at TNM I stage was closely associated with the risk of recurrence and shorter survival time (OS, p = 0.0042; TTR, p = 0.0500). Thus, our results have exhibited the potential value of KMO in predicting the risk of recurrence and patient survival in subgroups with normal AFP levels (AFP < 20 ng/ml) or in the early-stage HCC group, which would have been difficult using currently clinically available surrogate biomarkers.

Molecular mechanisms underlying recurrence or metastasis of HCC are very complicated. Genetically, chromosomal copy number alterations, such as the loss of alleles on 16q and the amplification of 1q, have been found to associate with HCC metastasis^31^,^32^. Recently, multiple genes associated with HCC progression and metastasis have also been identified, including PGE2^33^, Keratin 19^34^, FoxQ1^35^, ICAT^36^, and Gankyrin^37^. KMO locates in a pivotal position in KP, which is responsible for >95% of tryptophan degradation in mammals, ultimately leading to the formation of NAD^38^,^39^. NAD is a key regulator of several energy and signal transduction processes such as transcription, DNA repair, cell cycle progression, apoptosis and metabolism, which are relevant to tumor cell survival and proliferation. It functions as a coenzyme in metabolic pathways including glycolysis and the pentose phosphate pathway, thereby allowing the efficient production of NADPH, ribose-5-phosphate (Rib-5-P) and biosynthetic compounds used by the tumor for growth and angiogenesis^40^. Moreover, aberrant NAD metabolism is considered a hallmark of cancer: the high rates of aerobic glycolysis (Warburg effect) perturbs NAD metabolism, thereby leading to disrupt the cellular NADH/NAD^+^ redox homeostasis, and promote cancer progression^41^,^42^. Our data first exhibited that KMO expression was significantly upregulated in HCC tissues. As a pivotal enzyme in the kynurenine pathway, increased KMO expression might affect NAD concentration in HCC and then involved in HCC progression.

Previous studies showed that modulation of KMO activity was mainly involved in several neurodegenerative diseases, including Huntington's disease and Alzheimer's disease^6^,^43^. However, roles of KMO in tumor, including HCC, remain hitherto unknown. It has been previously reported that selective degradation of tryptophan created an immunosuppressive micromilieu of tumor both by depleting tryptophan and by accumulating immunosuppressive metabolites of the kynurenine pathway: On the one hand, tryptophan shortage inhibited T cells proliferation and causes a lack of accumulation of specific T cells at the tumor site^44^,^45^. On the other hand, the main tryptophan metabolites, such as Kynurenine, 3-hydroxykynurenine, and 3-hydroxyanthranilic acid, could suppress the T cell response and kill T cells, B cells and natural killer (NK) cells^46^,^47^. In this study, we found that KMO expression positively regulated proliferation, migration and invasion of HCC cells *in vitro*. Although underlying mechanism remains to be further investigated, our present study provides a clue for biological function of KMO in HCC.

To the best of our knowledge, this is the first study to report that high KMO expression is correlated with aggressive malignant phenotype of HCC. Our data indicate that KMO may serve as a novel prognostic marker for HCC and targeting KMO may provide a promising strategy for HCC treatment.

## Additional Information

**How to cite this article**: Jin, H. *et al*. Prognostic significance of kynurenine 3-monooxygenase and effects on proliferation, migration, and invasion of human hepatocellular carcinoma. *Sci. Rep*. **5**, 10466; doi: 10.1038/srep10466 (2015).

## Supplementary Material

Supplementary Information

## Figures and Tables

**Table 1:** Correlation between KMO expression and clinicopathologic parameters in HCC.

Variable (missing cases)		KMO protein		
	All cases	Low expression	High expression	*p*value
Age				0.886
≤50	104	68	36	
>50	101	67	34	
Sex				0.515
Male	174	113	61	
Female	31	22	9	
HBsAg				0.602
Negative	54	34	20	
Positive	151	101	50	
Serum AFP				0.316
≤20 ng/ml	74	52	22	
>20 ng/ml	131	83	48	
Liver cirrhosis				0.932
No	20	13	7	
Yes	185	122	63	
TNM				0.299
I	59	42	17	
II	120	79	41	
III–IV	26	14	12	
Child-Pugh class				0.777
A	195	128	67	
B	10	7	3	
Tumor size				0.268
≤3 cm	25	14	11	
>3 cm	180	121	59	
Tumor number				0.118
Single	162	111	51	
Multiple	43	24	19	
Tumor differentiation(3)				0.004*
I-II	37	32	5	
III-IV	165	102	63	
Vascular invasion				0.155
No	69	50	19	
Yes	136	85	51	

Chi-square test for comparison between groups. *p < 0.05. HBsAg, hepatitis B surface antigen; AFP, alpha-fetoprotein; TNM, tumor-node-metastasis

**Table 2:** Univariate and multivariate analysis of different prognostic parameters in patients with HCC by Cox regression analysis.

Factors	OS				TTR			
		Multivariate				Multivariate		
	Univariate p	HR	95%Cl		Univariate p	HR	95% Cl	*p*
Age: ≤50 vs >50	0.520	NA	NA	NA	0.420	NA	NA	NA
Sex: Male vs Female	0.244	NA	NA	NA	0.192	NA	NA	NA
HBsAg: Negative vs Positive	**0.028**	NA	NA	NA	0.168	NA	NA	NA
Serum AFP (ng/ml): ≤20 vs >20	0.215	NA	NA	NA	0.754	NA	NA	NA
Liver Cirrhosis : No vs Yes	0.307	NA	NA	NA	0.442	NA	NA	NA
TNM: I vs II vs III–IV	**0.000**	NA	NA	NA	**0.001**	1.633	1.216–2.192	**0.001**
Child-Pugh: A vs B	0.616	NA	NA	NA	0.509	NA	NA	NA
Tumor Size: ≤3 cm vs >3 cm	0.159	NA	NA	NA	0.774	NA	NA	NA
Tumor Number: Single vs Multiple	**0.003**	1.827	1.200–2.780	**0.005**	**0.010**	NA	NA	NA
Tumor Differentiation: I-II vs III-IV	**0.041**	NA	NA	NA	0.755	NA	NA	NA
Microvascular Invasion: No vs Yes	**0.000**	2.182	1.439–3.308	**0.000**	**0.035**	NA	NA	NA
KMO: Negative vs Positive	**0.001**	1.700	1.161–2.489	**0.006**	**0.003**	1.763	1.193–2.606	**0.004**

## References

[b1] Jemal A. Global cancer statistics. CA Cancer J Clin 61, 69–90 (2011). 21296855 10.3322/caac.20107

[b2] Sasaki Y. Risk of recurrence in a long-term follow-up after surgery in 417 patients with hepatitis B- or hepatitis C-related hepatocellular carcinoma. Ann Surg 244, 771–780 (2006). 17060771 10.1097/01.sla.0000225126.56483.b3 PMC1856577

[b3] Bruix J., Gores G. J. & Mazzaferro V. Hepatocellular carcinoma: clinical frontiers and perspectives. Gut 63, 844–855 (2014). 24531850 10.1136/gutjnl-2013-306627 PMC4337888

[b4] Thomas M. B. & Zhu A. X. Hepatocellular carcinoma: the need for progress. J Clin Oncol 23, 2892–2899 (2005). 15860847 10.1200/JCO.2005.03.196

[b5] Ueno M. Adjuvant chemolipiodolization reduces early recurrence derived from intrahepatic metastasis of hepatocellular carcinoma after hepatectomy. Ann Surg Oncol 18, 3624–3631 (2011). 21614626 10.1245/s10434-011-1800-6

[b6] Zwilling D. Kynurenine 3-monooxygenase inhibition in blood ameliorates neurodegeneration. Cell 145, 863–874 (2011). 21640374 10.1016/j.cell.2011.05.020 PMC3118409

[b7] Campesan S. The kynurenine pathway modulates neurodegeneration in a Drosophila model of Huntington's disease. Curr Biol 21, 961–966 (2011). 21636279 10.1016/j.cub.2011.04.028 PMC3929356

[b8] Schwarcz R., Bruno J. P., Muchowski P. J. & Wu H. Q. Kynurenines in the mammalian brain: when physiology meets pathology. Nat Rev Neurosci 13, 465–477 (2012). 22678511 10.1038/nrn3257 PMC3681811

[b9] Heyes M. P., Saito K. & Markey S. P. Human macrophages convert L-tryptophan into the neurotoxin quinolinic acid. Biochem J 283 (**Pt 3**), 633–635 (1992). 1534219 10.1042/bj2830633 PMC1130930

[b10] De Castro F. T., Brown R. R. & Price J. M. The intermediary metabolism of tryptophan by cat and rat tissue preparations. J Biol Chem 228, 777–784 (1957). 13475359

[b11] Guillemin G.J. Kynurenine pathway metabolism in human astrocytes: a paradox for neuronal protection. J Neurochem 78, 842–853 (2001). 11520905 10.1046/j.1471-4159.2001.00498.x

[b12] Giorgini F. Histone deacetylase inhibition modulates kynurenine pathway activation in yeast, microglia, and mice expressing a mutant huntingtin fragment. J Biol Chem 283, 7390–7400 (2008). 18079112 10.1074/jbc.M708192200

[b13] Filippini P. Emerging concepts on inhibitors of indoleamine 2,3-dioxygenase in rheumatic diseases. Curr Med Chem 19, 5381–5393 (2012). 22963664 10.2174/092986712803833353

[b14] Platten M., Litzenburger U. & Wick W. The aryl hydrocarbon receptor in tumor immunity. Oncoimmunology 1, 396–397 (2012). 22737628 10.4161/onci.19071 PMC3382870

[b15] Liu X., Newton R. C., Friedman S. M. & Scherle P. A. Indoleamine 2,3-dioxygenase, an emerging target for anti-cancer therapy. Curr Cancer Drug Targets 9, 938–952 (2009). 20025603 10.2174/156800909790192374

[b16] Edge S. B. & Compton C. C. The American Joint Committee on Cancer: the 7th edition of the AJCC cancer staging manual and the future of TNM. Ann Surg Oncol 17, 1471–1474 (2010). 20180029 10.1245/s10434-010-0985-4

[b17] Bruix J. Clinical management of hepatocellular carcinoma. Conclusions of the Barcelona-2000 EASL conference. European Association for the Study of the Liver. J Hepatol 35, 421–430 (2001). 10.1016/s0168-8278(01)00130-1 11592607

[b18] Jin G. Z. iTRAQ-2DLC-ESI-MS/MS based identification of a new set of immunohistochemical biomarkers for classification of dysplastic nodules and small hepatocellular carcinoma. J Proteome Res 10, 3418–3428 (2011). 21631109 10.1021/pr200482t

[b19] Wang C., A multicenter randomized controlled trial of percutaneous cryoablation versus radiofrequency ablation in hepatocellular carcinoma. Hepatology (2014). 10.1002/hep.27548 25284802

[b20] Llovet J. M. Liver cancer: time to evolve trial design after everolimus failure. Nat Rev Clin Oncol 11, 506–507 (2014). 25091613 10.1038/nrclinonc.2014.136 PMC12261308

[b21] Lee J. S. A novel prognostic subtype of human hepatocellular carcinoma derived from hepatic progenitor cells. Nat Med 12, 410–416 (2006). 16532004 10.1038/nm1377

[b22] Asahina Y. alpha-fetoprotein levels after interferon therapy and risk of hepatocarcinogenesis in chronic hepatitis C. Hepatology 58, 1253–1262 (2013). 23564522 10.1002/hep.26442

[b23] Huang J. Exome sequencing of hepatitis B virus-associated hepatocellular carcinoma. Nat Genet 44, 1117–1121 (2012). 22922871 10.1038/ng.2391

[b24] Shang S. Identification of osteopontin as a novel marker for early hepatocellular carcinoma. Hepatology 55, 483–490 (2012). 21953299 10.1002/hep.24703 PMC3914762

[b25] Shen Q. Serum DKK1 as a protein biomarker for the diagnosis of hepatocellular carcinoma: a large-scale, multicentre study. Lancet Oncol 13, 817–826 (2012). 22738799 10.1016/S1470-2045(12)70233-4

[b26] Gu X. High expression of MAGE-A9 correlates with unfavorable survival in hepatocellular carcinoma. Sci Rep 4, 6625 (2014). 25315972 10.1038/srep06625 PMC4197415

[b27] Li T. Risk factors, prognosis, and management of early and late intrahepatic recurrence after resection of primary clear cell carcinoma of the liver. Ann Surg Oncol 18, 1955–1963 (2011). 21240562 10.1245/s10434-010-1540-z

[b28] Peng S. Y. High alpha-fetoprotein level correlates with high stage, early recurrence and poor prognosis of hepatocellular carcinoma: significance of hepatitis virus infection, age, p53 and beta-catenin mutations. Int J Cancer 112, 44–50 (2004). 15305374 10.1002/ijc.20279

[b29] Trevisani F. Serum alpha-fetoprotein for diagnosis of hepatocellular carcinoma in patients with chronic liver disease: influence of HBsAg and anti-HCV status. J Hepatol 34, 570–575 (2001). 11394657 10.1016/s0168-8278(00)00053-2

[b30] Toyoda H. Clinical utility of highly sensitive Lens culinaris agglutinin-reactive alpha-fetoprotein in hepatocellular carcinoma patients with alpha-fetoprotein<20 ng/mL. Cancer Sci 102, 1025–1031 (2011). 21244578 10.1111/j.1349-7006.2011.01875.x

[b31] Tsuda H. Allele loss on chromosome 16 associated with progression of human hepatocellular carcinoma. Proc Natl Acad Sci U S A 87, 6791–6794 (1990). 2168560 10.1073/pnas.87.17.6791 PMC54623

[b32] Jia D. Genome-wide copy number analyses identified novel cancer genes in hepatocellular carcinoma. Hepatology 54, 1227–1236 (2011). 21688285 10.1002/hep.24495

[b33] Bai X. Prostaglandin E2 stimulates beta1-integrin expression in hepatocellular carcinoma through the EP1 receptor/PKC/NF-kappaB pathway. Sci Rep 4, 6538 (2014). 25289898 10.1038/srep06538 PMC5377465

[b34] Govaere O. Keratin 19: a key role player in the invasion of human hepatocellular carcinomas. Gut 63, 674–685 (2014). 23958557 10.1136/gutjnl-2012-304351 PMC3963546

[b35] Xia L., Forkhead box Q1 promotes hepatocellular carcinoma metastasis by transactivating ZEB2 and VersicanV1 expression. Hepatology 59, 958–973 (2014). 24005989 10.1002/hep.26735

[b36] Zhang Y. MiR-424-5p reversed epithelial-mesenchymal transition of anchorage-independent HCC cells by directly targeting ICAT and suppressed HCC progression. Sci Rep 4, 6248 (2014). 25175916 10.1038/srep06248 PMC4150107

[b37] Jing H. Gradually elevated expression of Gankyrin during human hepatocarcinogenesis and its clinicopathological significance. Sci Rep 4, 5503 (2014). 24999092 10.1038/srep05503 PMC4083285

[b38] Thevandavakkam M. A., Schwarcz R., Muchowski P. J. & Giorgini F. Targeting kynurenine 3-monooxygenase (KMO): implications for therapy in Huntington's disease. CNS Neurol Disord Drug Targets 9, 791–800 (2010). 20942784 10.2174/187152710793237430

[b39] Leklem J. E. Quantitative aspects of tryptophan metabolism in humans and other species: a review. Am J Clin Nutr 24, 659–672 (1971). 4253043 10.1093/ajcn/24.6.659

[b40] Chiarugi A., Dölle C., Felici R. & Ziegler M. The NAD metabolome--a key determinant of cancer cell biology. Nat Rev Cancer 12, 741–752 (2012). 23018234 10.1038/nrc3340

[b41] Heikal A. A. Intracellular coenzymes as natural biomarkers for metabolic activities and mitochondrial anomalies. Biomark Med 4, 241–263 (2010). 20406068 10.2217/bmm.10.1 PMC2905054

[b42] DeBerardinis R. J., Lum J. J., Hatzivassiliou G. & Thompson C. B. The biology of cancer: metabolic reprogramming fuels cell growth and proliferation. Cell Metab 7, 11–20 (2008). 18177721 10.1016/j.cmet.2007.10.002

[b43] Giorgini F., Guidetti P., Nguyen Q., Bennett S. C. & Muchowski P. J. A genomic screen in yeast implicates kynurenine 3-monooxygenase as a therapeutic target for Huntington disease. Nat Genet 37, 526–531 (2005). 15806102 10.1038/ng1542 PMC1449881

[b44] Uyttenhove C. Evidence for a tumoral immune resistance mechanism based on tryptophan degradation by indoleamine 2,3-dioxygenase. Nat Med 9, 1269–1274 (2003). 14502282 10.1038/nm934

[b45] Munn D. H. Inhibition of T cell proliferation by macrophage tryptophan catabolism. J Exp Med 189, 1363–1372 (1999). 10224276 10.1084/jem.189.9.1363 PMC2193062

[b46] Terness P. Inhibition of allogeneic T cell proliferation by indoleamine 2,3-dioxygenase-expressing dendritic cells: mediation of suppression by tryptophan metabolites. J Exp Med 196, 447–457 (2002). 12186837 10.1084/jem.20020052 PMC2196057

[b47] Iken K. Indoleamine 2,3-dioxygenase and metabolites protect murine lung allografts and impair the calcium mobilization of T cells. Am J Respir Cell Mol Biol 47, 405–416 (2012). 22517796 10.1165/rcmb.2011-0438OC PMC3488632

