# Supplementary material for: Prognostic significance of kynurenine 3-monooxygenase and effects on proliferation, migration, and invasion of human hepatocellular carcinoma
Source: Sci Rep. 2015 Jun 23;5:10466. doi: 10.1038/srep10466 (PMC4479133; doi:10.1038/srep10466)
Supplement: Supplementary Information [file srep10466-s1.doc]

**Prognostic significance of kynurenine 3-monooxygenase and effects on proliferation, migration, and invasion of human hepatocellular carcinoma.**

Haojie Jin^1*^, Yurong Zhang^1*^, Haiyan You^1*^, Xuemei Tao^1^, Cun Wang^1^, Guangzhi Jin^2^, Ning Wang^1^,

Haoyu Ruan^1^, Dishui Gu^1^, Xisong Huo^1^, Wenming Cong^2^ & Wenxin Qin^1^.

^1^ State Key Laboratory of Oncogenes and Related Genes, Shanghai Cancer Institute, Renji Hospital,

Shanghai Jiao Tong University School of Medicine

^2^ Department of Pathology, Eastern Hepatobiliary Surgery Hospital, Second Military Medical University

**Supplementary Figure**


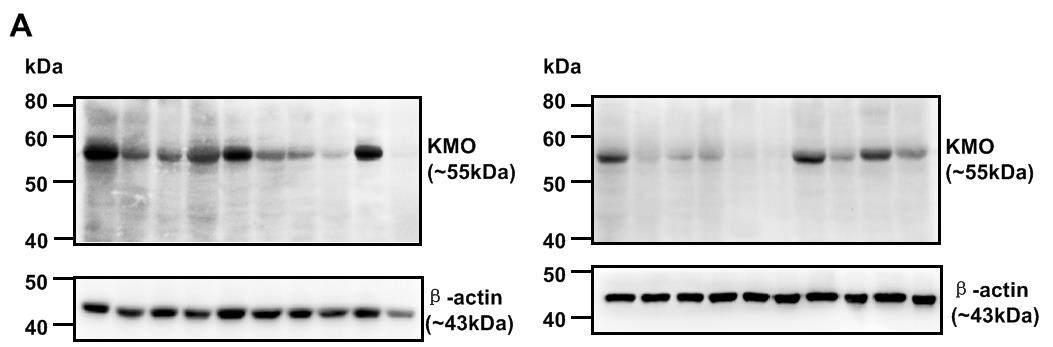


**Supplementary Figure 1.** Full-length images of Western blots results in Figure 1A.


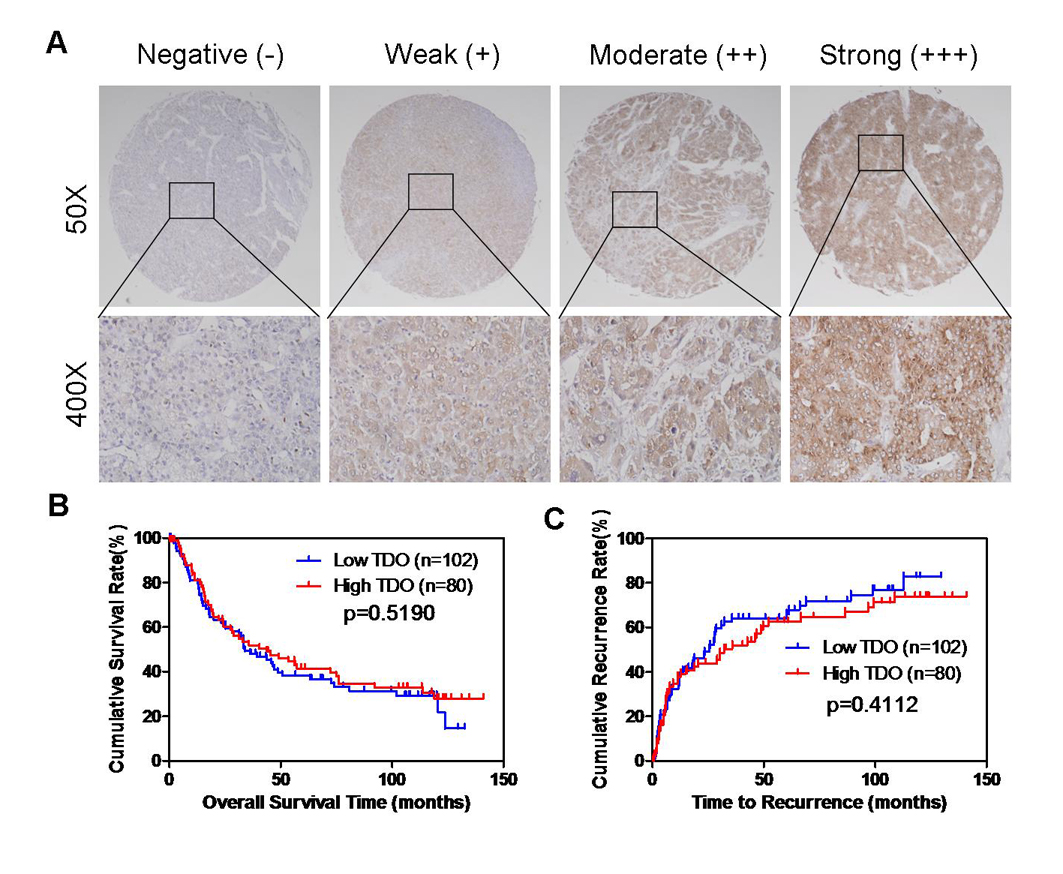


**Supplementary Figure 2. Correlation of TDO expression with OS and TTR in HCC patients.** **(A)** Representative photomicrographs showed negative (-), weak (+), moderate (++), or strong (+++) immunostaining of TDO in HCC specimens (magnification, ×50, ×400). **(B, C)** Kaplan-Meier curves of OS **(B)** and TTR **(C)** in 182 HCC patients.


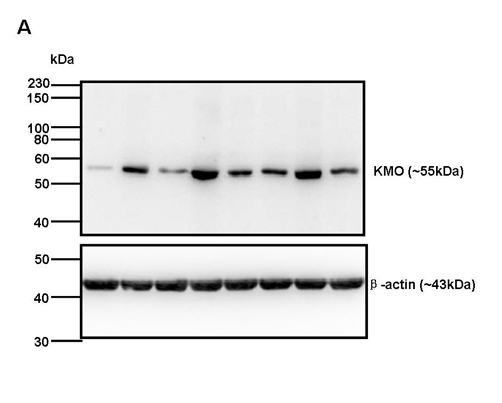


**Supplementary Figure 3.** Full-length images of Western blots results in Figure 5A.


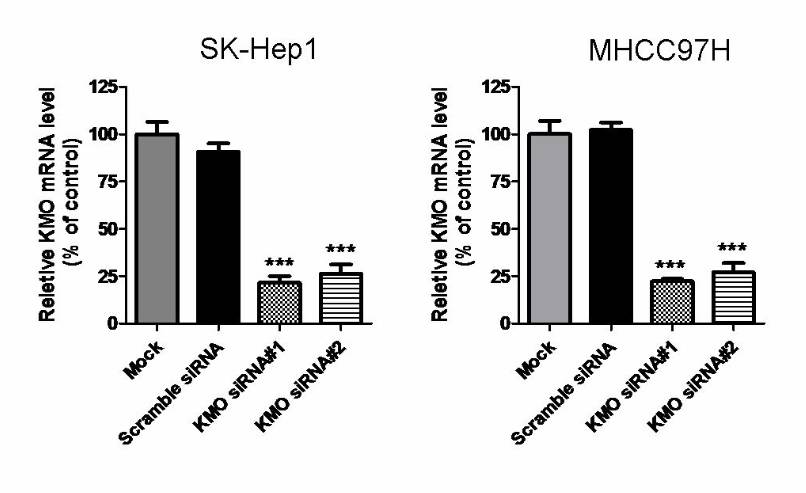


**Supplementary Figure 4. Knockdown of KMO in SK-Hep1 and MHCC-97H was verified by qRT-PCR, respectively.** SK-Hep1 and MHCC-97H cells were transfected with no siRNA (Mock),siRNA control (Scramble siRNA) or siRNAs against KMO (KMO siRNA#1 and KMO siRNA#2), respectively. Knockdown efficiency of KMO was verified by qRT-PCR. (***p< 0.001)


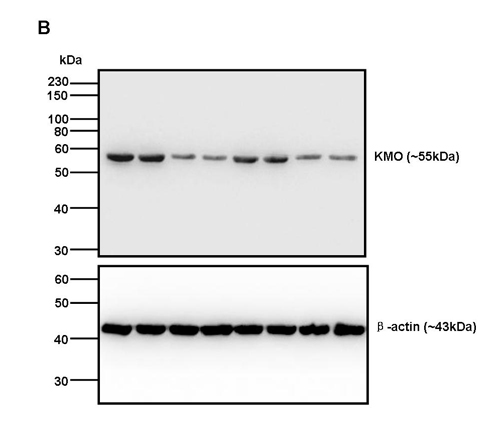


**Supplementary Figure 5.** Full-length images of Western blots results in Figure 5B.


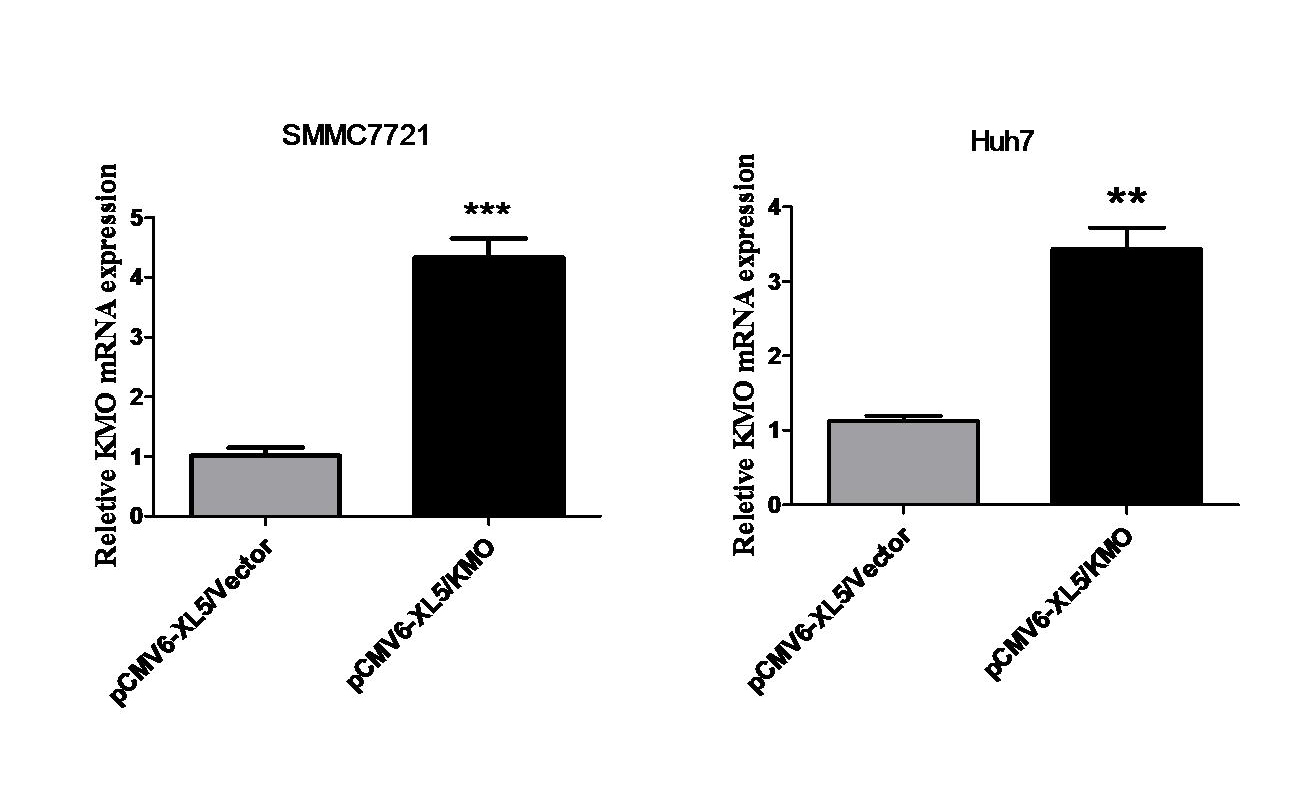


**Supplementary Figure 6. Overexpression of KMO in SMMC7721 and Huh7 was verified by qRT-PCR, respectively.** Overexpression of KMO in SMMC7721 and Huh7 cells transfected with pCMV6-XL5/Vector or pCMV6-XL5/KMO was verified by qRT-PCR., respectively.

**
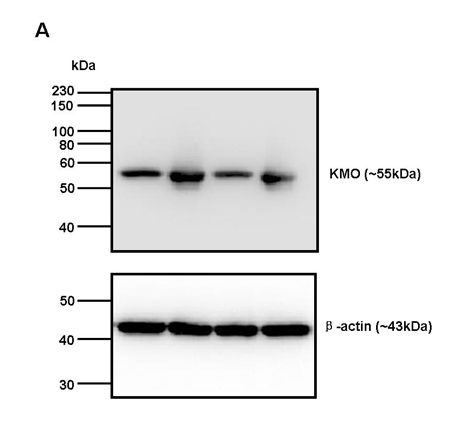
**

**Supplementary Figure 7.** Full-length images of Western blots results in Figure 6A.
